# Applications of Microfluidics and Organ-on-a-Chip in Cancer Research

**DOI:** 10.3390/bios12070459

**Published:** 2022-06-27

**Authors:** Sagar Regmi, Chetan Poudel, Rameshwar Adhikari, Kathy Qian Luo

**Affiliations:** 1 Department of Pharmacology, School of Medicine, Case Western Reserve University, Cleveland, OH 44106, USA; sagar005@e.ntu.edu.sg; 2 Department of Physics, Kathmandu University, Dhulikhel 45200, Nepal; chetan.poudel490311@gmail.com; 3 Research Centre for Applied Science and Technology (RECAST), Tribhuvan University, Kathmandu 44600, Nepal; rameshwar.adhikari@cdc.tu.edu.np; 4 Nepal Academy of Science and Technology (NAST), Khumaltar, Lalitpur 44700, Nepal; 5 Faculty of Health Sciences, University of Macau, Taipa, Macau, China; 6 Ministry of Education Frontiers Science Center for Precision Oncology, University of Macau, Taipa, Macau, China

**Keywords:** microfluidics, organ-on-a-chip, microfabrication, tumor microenvironment, circulating tumor cells, metastasis

## Abstract

Taking the life of nearly 10 million people annually, cancer has become one of the major causes of mortality worldwide and a hot topic for researchers to find innovative approaches to demystify the disease and drug development. Having its root lying in microelectronics, microfluidics seems to hold great potential to explore our limited knowledge in the field of oncology. It offers numerous advantages such as a low sample volume, minimal cost, parallelization, and portability and has been advanced in the field of molecular biology and chemical synthesis. The platform has been proved to be valuable in cancer research, especially for diagnostics and prognosis purposes and has been successfully employed in recent years. Organ-on-a-chip, a biomimetic microfluidic platform, simulating the complexity of a human organ, has emerged as a breakthrough in cancer research as it provides a dynamic platform to simulate tumor growth and progression in a chip. This paper aims at giving an overview of microfluidics and organ-on-a-chip technology incorporating their historical development, physics of fluid flow and application in oncology. The current applications of microfluidics and organ-on-a-chip in the field of cancer research have been copiously discussed integrating the major application areas such as the isolation of CTCs, studying the cancer cell phenotype as well as metastasis, replicating TME in organ-on-a-chip and drug development. This technology’s significance and limitations are also addressed, giving readers a comprehensive picture of the ability of the microfluidic platform to advance the field of oncology.

## 1. Introduction and Overview

### 1.1. Introduction to Microfluidic Technology

Microfluidics is a multidisciplinary branch of science that studies the behavior, control, and manipulation of fluids at the micron scale (one-millionth of a meter), employing microminiaturized devices with chambers and very small channels with diameters ranging from tens to hundreds of micrometers [1]. The physics of fluids differs dramatically at the microscale, resulting in its specific characteristics in scientific discovery [2]. Microfluidics, which emerged in the 1980s, has found applications in the creation of inkjet printers, lab-on-a-chip, and organ-on-a-chip technology, as well as chemical synthesis, cell analysis, and medication administration.

**Figure 1:**
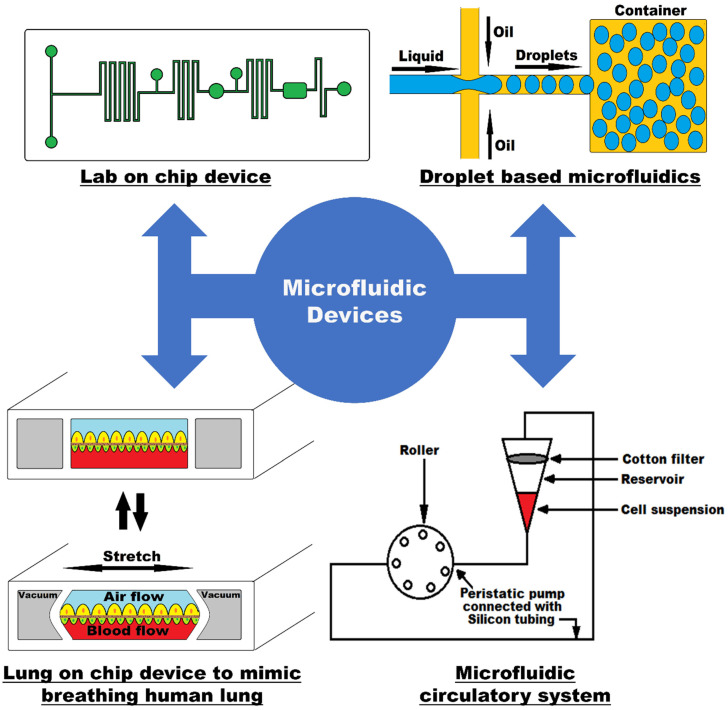
Microfluidic devices showing lab-on-a-chip, droplet-based microfluidics, organ-on-a-chip, and circulatory system that can produce the shear stress mimicking the human physiological system.

Microfluidic technology was first applied in the analysis as it offers many capabilities such as the usage of smaller amount of reagent, performance with high resolution and sensitivity, minimal cost, and less analysis time. Moreover, it exploits the small channel size for low Reynold’s number laminar flow, hence giving a controlled environment for molecular concentrations in space and time [1]. One of the key consequences of having a laminar flow inside the microfluidic channels is that because co-flowing fluids do not mix in the usual sense, molecular transfer between them is frequently accomplished by diffusion [3]. The drawings in Figure 1 show different modalities of a typical microfluidic device.

Microfluidic devices are made up of engraved microchannels that are linked to the macroenvironment through holes through which fluid can be injected and expelled from the channels through embedding with Quake valves and a pressure control system [4]. These small devices, which are only a few centimeters in size, provide several advantages over real-scale systems, including the ability to analyze samples or reagents with less volume, making it more cost efficient, decreasing the experiment time, and having a small footprint. A “lab-on-a-chip” is a miniature device that combines high-resolution laboratory procedures for fluid transport, mixing and analysis into a system that fits on a chip [5]. Another type of microfluidic device is an “organ-on-a-chip,” which duplicates the operation of a living organ inside a chip and allows for research mimicking the human physiology [6]. These are used to replicate the functionalities of a human organ, among which we can find the models of heart-on-a-chip, lung-on-a-chip, liver-on-a-chip, tumor-on-a-chip, and others.

**Figure 2:**
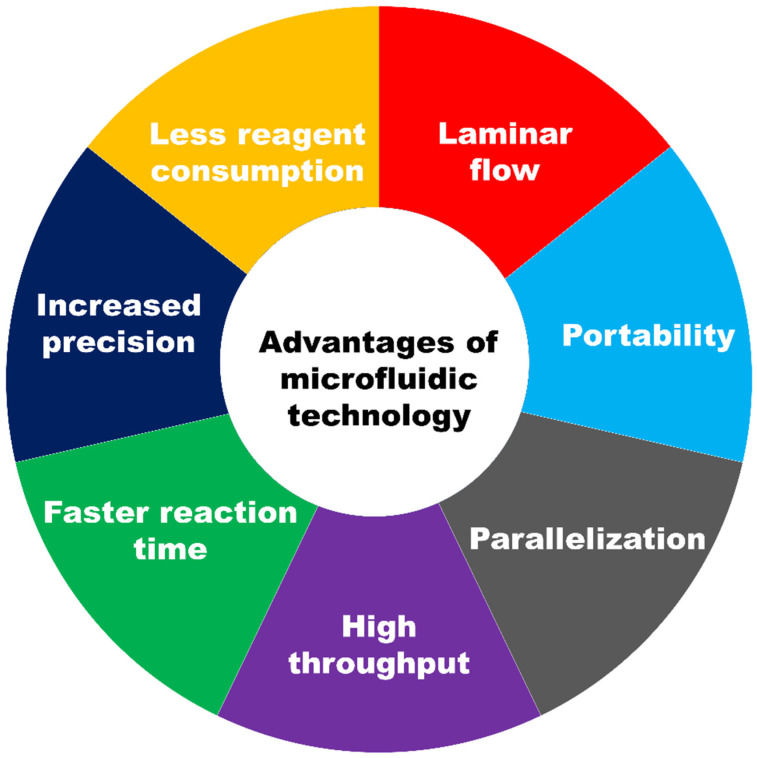
Schematic showing various advantages of microfluidic technology.

The chief advantages of the microfluidic device are summarized in Figure 2.

To begin with, due to the small dimension of the fabricated device, generally in the range of a few centimeters by a few centimeters, the cost for the batch production is minimal. The consumption of a reagent in microfluidics devices is drastically reduced (approximately 10^2^–10^3^ less sample volume), lowering the overall cost in the application sector. In microchannels, the low Reynold number ensures that fluid flow and chemical transfer are predictable and orderly. The device’s size, often in the order of a few centimeters, makes it portable, expanding the number of applications it can be used for, including point-of-care. The parallelization feature in microfluidics allows numerous assays to be multiplexed on a single chip. The simultaneous processing of numerous analytes also significantly reduces experiment time, resulting in high throughput. The carved microchannels’ customized architecture allows for the improved control of experimental conditions, resulting in more precise and accurate results. A microfluidic system also offers a faster reaction time due to the lower diffusion of any solute in the microchannels. Due to these advantages of using a microfluidic platform, they have been used in a range of application areas.

### 1.2. Historical Developments of Microfluidics

**Figure 3:**
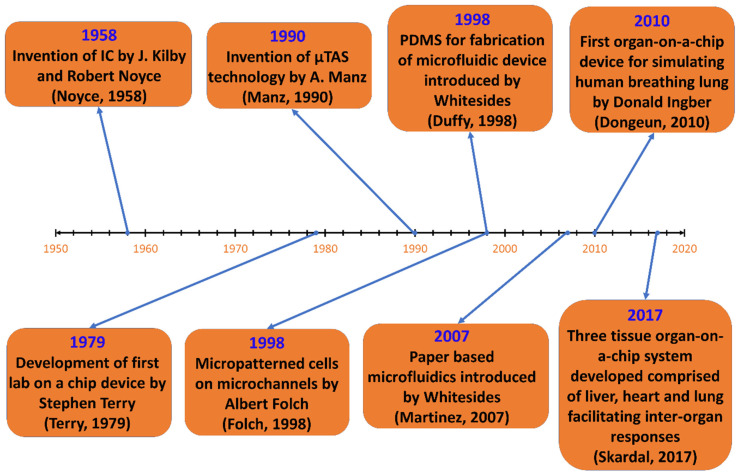
Schematic representation showing the historical development of microfluidic devices.

The roots of microfluidics come from the microelectronic industry dating back to the 1950s. The invention and development of the transistor revolutionized the electronics industry, casting away the previously used components for electronic devices. While chemists and biologists looked for ways to miniaturize their analytical methods, the microelectronics industry used photolithography, etching, and bonding techniques to improve its silicon-based micromachining process. Thus, the merging of bioanalytical and microelectronic fields can be considered as the birth of microfluidics. So, we can say that the development of microfluidic technology is made possible by using methods and materials from several other areas. Figure 3 illustrates the historical development of microfluidic devices to this date.

In 1949, Werner Jacobi, a German engineer, carried out the first development of integrated circuits (ICs). Later, Jack Kilby, an American engineer in 1958, took upon the challenge to create components of uniform size and shape with integrated wires that could be easily assembled to make circuits. He then showed the first working germanium-based ICs which were later made up of silicon by Robert Noyce, an American physicist [7].

Photolithography, the process used in microfabrication to pattern parts on a thin film, was developed by Jay W. Lathrop and James R. Nall, who were given this assignment by the US military in 1952, to find a way of reducing the electronic circuits to fit in the limited space of proximity fuse. Using the chemical photoresist, they patterned the germanium. Thus, they successfully created a 2D miniaturized hybrid integrated circuit with transistors using this technique. They wrote a paper and patented their discovery in 1958 [8]. Later, nonlithographic techniques were employed in microfabrication as photolithography turned out to be a difficult process while working with nonsemiconductors. Researchers went on a hunt to find a technique that could be used in a wide range of materials, is less expensive and capable of 3D patterning the substrates. Then, the method of soft lithography, the technique for fabricating and replicating structures using elastomeric molds, was born.

In the 1970s, Stephen Terry from Stanford University developed a silicon wafer-based miniature gas chromatography (GC) analyzer that includes an injection mechanism, gas supply, capillaries, and output miniaturized thermal conductivity detector. The microfabrication of the detector was carried out similarly as the IC processing methods [9]. This was the first example of a “Lab-on-a-chip”. In biomedical fields, many researchers investigated the development of microelectromechanical systems (MEMS), in the 1990s, in search of a method for the management and control of fluid in microchannels, which contributed to the development of microfluidics.

Polydimethylsiloxane (PDMS) is a polymer commonly utilized in microfluidic device fabrication. F.S. Kipping, who carried out the majority of the early work on silicon polymers, characterized siloxanes, the macromolecules that make up PDMS’ foundation [10]. The early microfluidic devices built of silicon and glass had some limitations, such as being pricey and having low gas permeability, making them unsuitable for biological investigations. A novel material was required for the manufacturing of a microfluidic chip that was transparent, flexible, inexpensive, and simple to use. George Whitesides, in the late 1990s, introduced a way of manufacturing low-cost microfluidics using PDMS. PDMS might be blended with a cross-linking agent and put into microstructure molds before being heated to create an elastomeric replica [11]. As a result of its transparency, deformability, low cost, simple molding, and gas permeability, PDMS has become a new desirable material for the fabrication of microfluidic devices.

An “organ-on-a-chip” is a microscale mimic of a human organ or body. Employing the physiological and mechanical conditions experienced in a body, the aim is to use it in tissue modelling and drug testing [12]. In the late 1990s, microfluidic devices had been created for cell biology applications in patterning cells and proteins. Albert Folch and Mehmet Toner were able to produce micropatterns of cells on tissue culture substrates in a network of deep elastomeric microchannels using collagen and fibronectin as templates [13]. Following this, researchers began working on tissue and organ prototypes for drug trials and pathophysiological research. Later, many multi-channel organ-on-a-chips of brain, lung, heart, kidney, artery, skin, bone and cartilage have been proposed that can simulate the activities and physiological response of the entire organ.

### 1.3. How Microfluidic Devices Work

**Figure 4:**
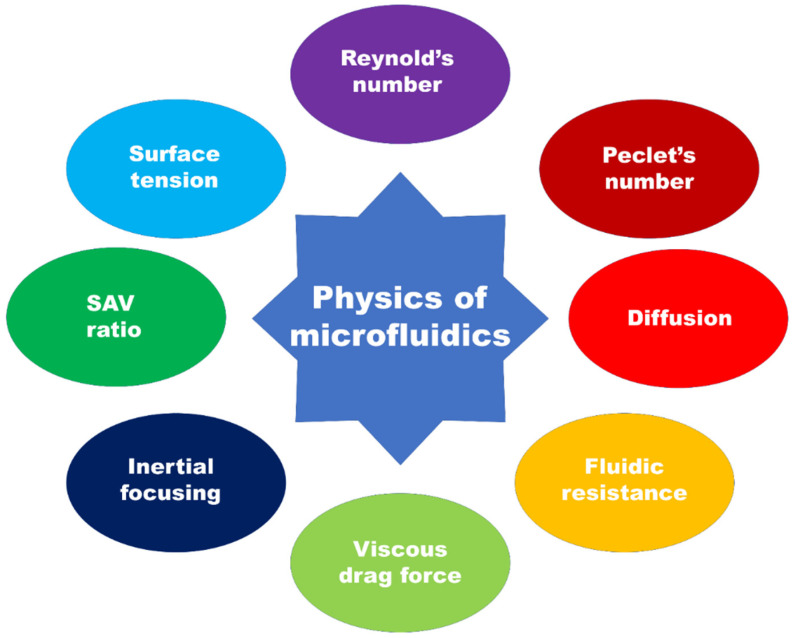
Illustration showing the basic principle of microfluidics in relation to various physical phenomena.

Microfluidics is the branch of science and technology (specifically fluid dynamics) which deals with the control and manipulation of fluid at a microscale. By exploiting the properties that the fluid will exhibit at this scale as illustrated in Figure 4, one can perform reactions, analyses, and investigations in the fields of biological, chemical, environmental science, etc.

The fluid motion is modelled by Navier–Stoke’s equation, the equation that governs the fluid flow. Considering the incompressible Newtonian fluid, the Navier–Stoke’s equation is given by

$$\rho \left[\frac{\partial v}{\partial t}+\left(v\cdot \nabla \right)v\right]=\rho g-\nabla p+\eta \cdot v$$

where ***v*** is the velocity of the flow, *ρ* is the density of the fluid, *g* is the acceleration due to gravity, *p* is pressure and *η* is the viscosity. Here, the term on the left-hand side corresponds to the spatial variation of the velocity and the term on the right-hand side corresponds to volume, surface and viscous forces [2].

Microfluidic devices are fabricated with the channels having diameters ranging from 100 nm up to 100 μm, which greatly alters the surface to volume ratio. The low Reynold’s number characterizes the flow as laminar, whereas the Peclet number is high, which leads to unique microfluidic mixing regimes. At the micrometer scale, the dominant forces are different from those at the macroscale and the flow exhibits different properties that can be mathematically modelled. The order of parameters on the microscale is generally width and height from 1 to 100 μm, flow rates of nL s^−1^–μL s^−1^ and volume in the order of pL–μL.

#### 1.3.1. Reynold’s Number

The flow regime of fluid flow is characterized by Reynold’s number. It is a dimensionless number which quantifies the inertial relative to viscous forces and gives insight into whether the fluid flow is laminar or turbulent. The low Reynold number characterizes the dominance of viscous force over inertial force and in high Reynold’s number inertial force dominate [14]. This dimensionless quantity has a considerable effect on the flow profile of the fluid. Mathematically,

$$Re=\frac{\rho vL}{\eta }$$

where *ρ* is the density of fluid, *ν* is the characteristic velocity and *L* is the characteristic linear dimension and *η* is the fluid viscosity. The Reynold’s number in the microfluidic channel is reduced as its characteristic dimension is reduced and gives rise to regular and predictable laminar flow.

#### 1.3.2. Peclet’s Number

Peclet’s number is a dimensionless number which represents a ratio of the convection rate over diffusion rate and gives information on the mass transport of the fluid. A higher Peclet’s number characterizes the dominance of convection over diffusion, which results in high concentration gradients, and a low Peclet’s number means that diffusion dominates, resulting in a uniform concentration across the fluid.

$$Pe=\frac{vL}{D}$$

where *D* is the coefficient of diffusion

It can be observed that reducing the dimensions of the system reduces the Peclet number. Thus, convection does not prevail in a microfluidic system and mixing occurs only due to diffusion, whose rate is way slower than that of convective mixing. The platforms where quick mixing is not required and the cost of reagents are issues, making microfluidics an ideal tool. For example, a blood glucose meter only requires a small pint of blood to read the glucose concentration [15].

#### 1.3.3. Diffusion

Diffusion is the net movement of particles from a high concentration to low concentration driven by the concentration gradient and, over time, the average concentration of particles throughout the volume is the same. For one dimension, the average diffusion length can be expressed as d^2^ = 2Dt, where d is the distance a particle moves in time t and D is the diffusion coefficient of the particle [15]. Diffusion has a huge effect on the microscaled dimension for the squared distance. As a result, the particles take less time to diffuse in a microscale system.

#### 1.3.4. Fluidic Resistance

Fluidic resistance is defined as the ratio of pressure drop to volume flow. The flow rate of microchannel is given by Q = ΔP/R, where Q is the flow rate, P is the pressure drop across the channel and R is the channel resistance [15]. This is analogous to the electrical resistance relation with voltage and current in which the pressure drop is analogous to the voltage drop across the wire and the flow rate is equivalent to the flow rate [16].

#### 1.3.5. Viscous Drag Force

Drag force is the force exerted in the particle in a moving fluid which causes the particle to move along the direction of motion of the fluid. In laminar flow fluid motion in a microchannel, the flow regime is Stoke’s flow and the drag force is given by F_d_ = 6πrηv (for spherical particle), where v is the velocity of particle, η is the viscosity of the fluid and v is the relative velocity between fluid and particle [17].

#### 1.3.6. Inertial Focusing

Inertial microfluidics has gained a lot of popularity in recent years; it uses the inertial focusing method and hydrodynamic phenomena to sort out the particles based on their size. The balance between two or more forces in the channel causes particles (or bioparticles such as DNA, RNA, cells and proteins) to attain an equilibrium position in the microchannel and by controlling the flow rate, particles can be separated with high precision [18,19].

The separation in a spiral microchannel, for example, is achieved by balancing the inertial lift force and the dean drag force. There is a lateral migration perpendicular to the direction of fluid motion when the particle migrates to the microchannel, together with the drag of the particle in that direction. This causes the particle to migrate to an equilibrium location, resulting in inertial lift force, which induces lateral migration. The dean drag force occurs on particles travelling in a curved channel when the centripetal force on the curve causes a pressure gradient between the inner and outer sides of the wall, resulting in a secondary flow known as dean flow, which causes counter rotating dean vortices. The dean flow generates the dean drag force, which can be utilized to balance the inertial lift force and control the particle’s equilibrium location, which is useful for particle sorting [20].

So, as microfluidic technologies allow the controlled flow of fluids, they have been used in particle sorting, especially in biological research for the manipulation of bioparticles such as DNA, RNA, cells and proteins.

#### 1.3.7. Surface Area to Volume Ratio

Microscale devices have the characteristic of a high surface area to volume ratio (SAV), which makes the surface forces dominant over inertial forces. Digital microfluidic technology exploits this feature where droplets of water or liquid are created and transported in a controlled fashion for storage or analysis. High SAV makes capillary electrophoresis (CE) efficient by removing excess heat more rapidly. Microfluidics allows a greater dissipation of Joule heat which leads to increased performance, higher sensitivity, and the increased resolution of separations in CE instruments [21].

#### 1.3.8. Surface Tension

Surface tension is the tendency of a liquid surface to minimize its surface area. Capillary action is the ability of the liquid to flow upward in a narrow region without the aid of external forces. Since the microfluidic device has a dimension of tens to hundreds of microns, the capillary forces in the device become significant for capillary pumping [2,15].

### 1.4. Designing Materials for Microfluidics

**Figure 5:**
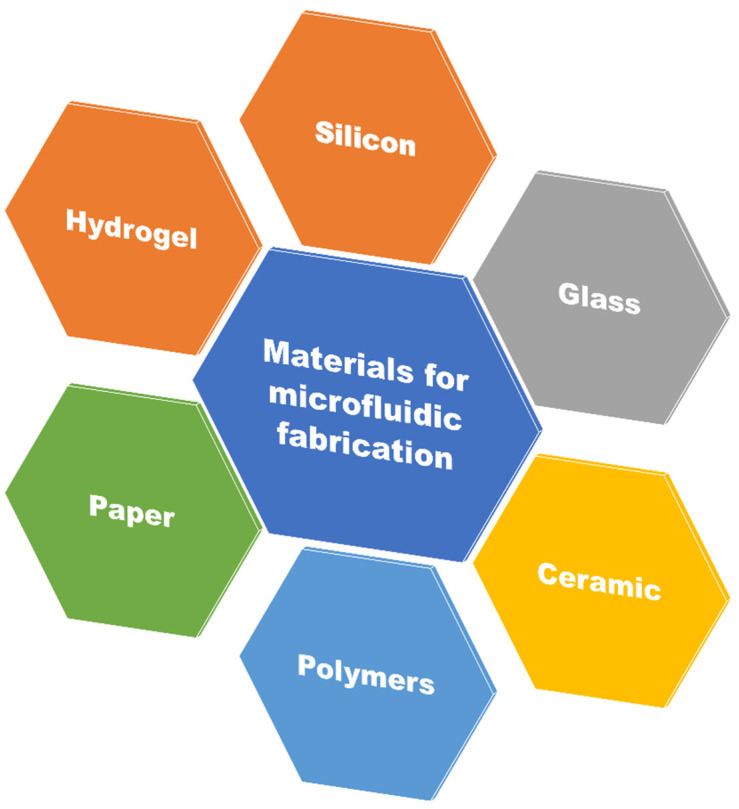
Scheme showing different classes of materials that were used for microfluidic fabrication.

Various methods can be employed in the fabrication of a microfluidic device such as etching, molding, lithographic technique, 3D printing in the design of chambers and channels on which holes are engraved to bridge the micro- and macroenvironment. Several materials have been used to manufacture microfluidic devices over the years such as polymers, glass, silicon and even papers depending upon the application area [22]. The material used in the manufacture of microfluidics would be inorganic materials (such as silicon, and glass), organic materials (such as paper) and polymers (such as PDMS, PMMA, and COC) as illustrated in Figure 5. Some of the commonly used materials for the manufacture of microfluidics are introduced below.

#### 1.4.1. Polydimethylsiloxane (PDMS)

Polymers were frequently employed in the production of a microfluidic device that was introduced several years after inorganic materials due to their ease of access, low cost, flexibility, and superior biochemical performance [23]. Polydimethylsiloxane (PDMS) has replaced expensive and time-consuming materials such as glass and silicon as the most extensively used and accepted material for manufacturing microfluidic devices. Transparency, flexibility, biocompatibility, and gas permeability set it apart from its traditional counterparts. The ability to make the desired device in a matter of hours with the cheap material is always an advantage in this research area, among which PDMS hails as one of the fundamental materials in microfluidics. Photolithographic or other processes are used to create device molds on which PDMS structures are cast which can be used to manufacture sophisticated microfluidic designs. The PDMS model may be covalently bonded to the glass substrate using a simple plasma process, resulting in a sealed microfluidic system [24].

The deformability of PDMS allows for leak-proof fluidic connections and the inclusion of valves in biological studies. The PDMS device is gas permeable, allowing gas to flow freely between the cells without the use of external air. Furthermore, by modifying its composition, its air permeability may be easily adjusted [25]. Its transparency aids in on-chip imaging as well. However, PDMS has a few drawbacks as well. The prime drawback is its hydrophobicity which leads to the creation of air bubbles in the channels and can absorb small hydrophobic molecules such as biomolecules and drugs in the solution in biological studies which ultimately bias the result for bio-experiments for which rising with ethanol before the experiment is suggested. The ageing of PDMS will alter its mechanical properties as well, and therefore it cannot be stored for a long time [26].

#### 1.4.2. Silicon

The first used material for the manufacture of microfluidics was silicon as it has advantages in its thermal conductivity and resistance to organic solvents. Because they are disposable, affordable, and easy to produce, they are an ideal contender for device fabrication. However, the material’s hardness and opacity made it unsuitable for microfluidic applications. The transparency problem makes fluorescence detection and fluid imaging challenging as silicon is transparent at the infrared scale but not in the visible range [23]. Furthermore, the valve and pump components are difficult to produce due to their hardness.

#### 1.4.3. Glass

After silicon, glass was the focus in manufacturing devices due to its similar advantages of silicon and moreover high optical transparency and high-pressure resistance, but the main shortcoming came down to high cost [27]. It is even used as an alternative to PDMS due to its low drug absorptivity. With high thermal conductivity along with stable electro-osmotic mobility on the surface, it outperforms other materials but due to its hardness and high fabrication cost, it led researchers to seek out alternative low-cost materials.

#### 1.4.4. Paper

Paper has recently gained popularity as a possible medium for microfluidic device construction due to its low cost, portability, and ability to be chemically manipulated through composition alterations or surface chemistry. Three-dimensional designs can easily be created using paper as multiple layers which can be stacked together. The capillary action in paper-based microfluidics pulls the solution through the device and has been used in biochemical, medical, and forensic analysis. Colorimetric, electrochemical and chemiluminescence can all be used to detect components; however, most paper microfluidic devices only use colorimetric detection [28]. Despite these advantages, the issues with paper-based microfluidics are the loss of mechanical strength in the wet state and the limitation on thickness to attain transparency.

## 2. Microfluidic Devices in Cancer Research

### 2.1. Fundamentals of Cancer Metastasis

The human body is composed of many tiny cells which coordinate among themselves to form tissue and organ. Cells grow and divide for a period and stop growing and only form new cells for replacing defective and dying cells. Cancer is a disease in which body cells grow uncontrollably and spread into other parts of the body. Unlike normal cells, cancer cells continue to grow and divide to form many more cells. Damage to cells’ DNA is the major cause of this abnormal growth of cells.

The replication of cancer cells forms a tumor which can be benign or malignant. A benign tumor is not harmful and does not spread, whereas a malignant tumor grows and spreads to other parts of the body by the process called metastasis. The diagnosis of cancer is carried out by lab tests, imaging tests or biopsy. In lab tests, blood, urine, or other bodily fluids are used to make a diagnosis. An imaging test creates a picture of areas inside of the body to find tumors. MRI, nuclear scan, PET scan, CT scan, bone scan, ultrasound, and X-rays are some methods or conducting an imaging test. Additionally, biopsy is a procedure in which doctors remove the sample of the tissue through excision or incision. Then, a pathologist observes the tissue under a microscope and runs the test to see if the tissue is cancerous.

**Figure 6:**
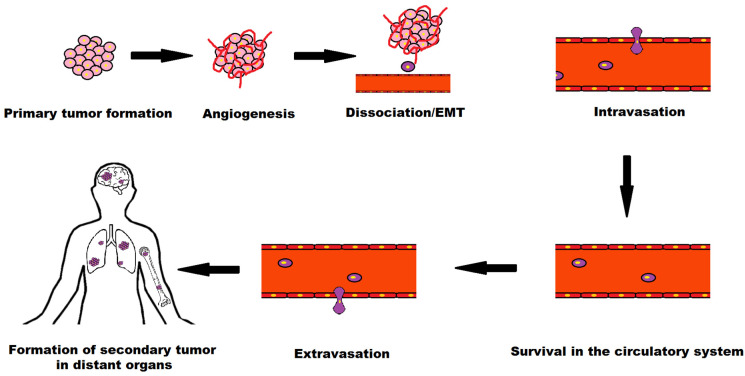
Schematic showing the process of cancer metastasis.

Metastasis is the process in which cancer cells leave the primary tumor site and travel to different sites through the circulatory or lymphatic system and form secondary tumors. The metastatic cascade is elucidated in Figure 6.

The key stages of metastatic process are (i) detachment of a tumor cell from the primary tumor site, (ii) invasion of basement membrane by extension or penetration through neighboring tissues, (iii) intravasation into the vascular or lymph vessels of the nearby region, (iv) survival as circulatory tumor cells (CTCs), (v) extravasation from blood vessels to secondary tissue and (vi) infiltration at subsequent tumor site [29].

Here, intravasation and extravasation are the entry and exit of tumor cells from the circulatory or lymphatic system. Circulatory tumor cells (CTCs) refer to the cells that are shed from the primary tumor site to the circulatory or lymphatic system and carried around the body in blood circulation. There are very few to be found in blood, around 1–10 CTCs per mL of whole blood [30].

### 2.2. Microfluidics in Cancer Research

For nearly two decades, scientists and researchers have been converging cancer research with microfluidic technology and developed several devices for research applications with the benefits of low cost, portability, small sample size, high sensitivity, and fast processing speed; thus, microfluidics has emerged as a promising tool for research and application in oncology.

Microfluidics platforms in cancer research allow a noninvasive cancer diagnosis. The biomarkers of cancer cells found in blood are DNA, mRNA, and CTCs; however, these biomarkers are present in very low levels (1–10 CTCs in 1 mL of whole blood [30]). As a result, researchers are looking into microchannel-based devices, which can carry out high-sensitivity separations. As a result of the great sensitivity of biomarkers, microfluidic technology has now been used in anti-cancer drug discovery.

Microfluidics provides an efficient platform for multiplexed analysis by allowing PCR, electrophoresis, and hybridization arrays to be performed on chips. Moreover, single-cell research and cell-to-cell variety in terms of drug response to diverse stimuli are possible using this technology. Therefore, by carefully manipulating concentration gradients, extracellular matrix (ECM) components and cell–cell analysis, microfluidics provides a greater picture of the cellular environment [31]. The Table 1 shows the approaches used for the inertial separation of CTCs.

**Table 1:** An overview of microfluidic tools used in cancer research.

Application of Microfluidic Technology in Oncology	Description	References
Isolation of CTCs	Performing label free and label-based methods for separation of cancer cells from background blood cells	[32,33,34,35,36,37,38,39,40]
Studying cancer cell phenotype	For studying the mechanical qualities that influence the migration of cancer cells and metastatic pattern	[41,42,43,44,45]
Studying shear stress	For characterizing the biophysical response of tumor cells due to shear stress in circulation	[46,47,48,49,50]
Studying metastasis	For studying the metastatic cascade by developing microfluidic tools able to reproduce biophysical, biomechanical and biochemical environment	[51,52,53,54,55,56]
Anti-cancer drug screening using droplet microfluidics	For allowing programmable drug absorption, confinement and controlled release	[57,58,59,60]
Replication of tumor microenvironment (TME) on chip	For recapitulating the key features of tumor microenvironment including tumor-stromal interaction, extracellular matrix (ECM) components, biophysical and metabolic factors	[61,62,63]
Studying angiogenesis and developing vascularized tumor on chip	For recreating prominent features of TME for oxygen and nutrient delivery to tumor cells	[64,65,66]
Organ-on-a-chip	For replicating the physiological aspects of an organ for replicating the structural, mechanical and biological factors for understanding cancer biology and advancing drug development process	[67,68,69,70]

Some application areas of microfluidics in the field of oncology are described in the following section.

#### 2.2.1. Microfluidic Device for Isolation of CTC

In the process of metastasis, cells are shed from the primary tumor site into the vasculature or lymphatics (intravasation) and carried around the body through blood circulation and can enter the distance organ to form a secondary site (extravasation). The cells are known as circulating tumor cells (CTCs). Metastasis is the leading cause of cancer-related death; thus, the detection and analysis of CTCs are of paramount importance to assist early diagnosis of patients and design appropriate treatments.

**Figure 7:**
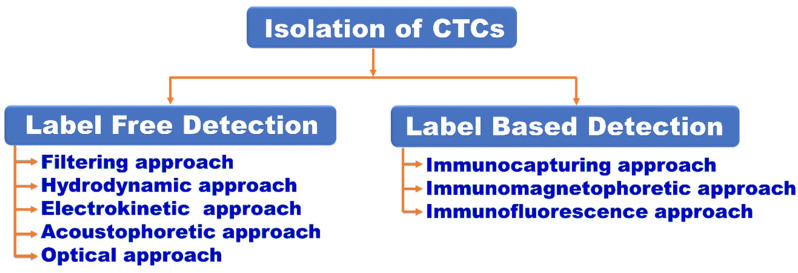
Scheme showing different ways of isolation of CTCs.

The number of CTCs in the peripheral blood stream has been discovered to be linked to the extent of cancer metastasis and progression. As a result, counting CTCs in the blood can help determine the stage of cancer growth [71]. Based on the biochemical and physical properties of CTC, microfluidics devices can isolate CTCs using various of methods, as mentioned in Figure 7.

CTCs can be captured using a variety of methods: filtration, centrifugation, magnetic separation, and others. Microfluidics integrates these methods into the microscale to achieve the separation and detection of CTCs. Furthermore, microfluidics has its advantages over the traditional method for small size, less sample, high sensitivity, and portability along the cellular size matching with the channel.

Based on biophysical or biochemical approaches, the isolation and detection of CTCs using microfluidics can be broadly classified into two categories: label-free detection and label-based detection.

The label-free detection method uses the properties of physical shape and size, density, deformability, dielectric properties and viscosity of CTCs. Micropores, micropillar arrays, deterministic lateral displacement, spiral channel, inertial focusing, acoustic waves, electro-kinesis and optical methods are the technologies developed using microfluidic devices for label-free detection. When compared to marker-based techniques for CTC trapping, label-free alternatives promise a higher yield [72] without compromising cell viability and gene expression profiles [31].

As CTC are larger than normal blood cells (size of blood cells: 5–15 μm; size of CTCs: 15–25 μm [73]), it is advantageous for size-based CTCs isolation process by either physical filtering or hydrodynamic forces. A physical filtering approach is a label-free method which can be modelled in micropores [32] and micropillars [33] for the high-throughput isolation of CTCs. On the other hand, a hydrodynamic isolation approach is based on hydrodynamic inertial effect in a continuous and precisely controlled flow due to the interaction between cell and obstacles in the microfluidic channels. Deterministic lateral displacement (DLD) [34], Microvortex [35], Dean flow fractionalization [36] and pinch flow [37] are the approaches used for the inertial separation of CTCs (Table 1).

An electrokinetic approach for the separation of cancer cell employs the electrical properties of cells to manipulate particles. Two broadly classified electrokinetic approaches are electrophoresis and dielectrophoresis. Electrophoresis is the effect where a particle having charge is induced to move in an electric field and dielectrophoresis (DEP) is an electrokinetic process in which a nonuniform electric field exerts force on a dielectric particle manipulating it to move in a high or low electric field gradient. Described by Herbert Pohl [74], it has been efficiently employed in CTCs isolation relying on the distinct dielectric properties between the cells. Mohommed et al. [38] used the technique to distinguish and separate human breast cancer cells (MCF-7) from colorectal cancer cells (HCT-116). Moon et al. [39] combined the inertial separation and DEP process to be benefited from both high throughput and an improved selectivity and efficiency of 99% was reported.

Similarly, in acoustophoretic approach of cancer cell isolation, sound waves are employed to move particles. When a suspended particle is subjected to an acoustic standing wave field, it will be affected by radiation force, which will cause particles to move in the sound field if the particle and surrounding medium have different acoustic properties. The size of the particle, the amplitude of the acoustic pressure, and the frequency of the sound wave all influence the particle’s velocity [75].

The optical technique for bioparticle manipulation uses optical force to isolate the target cells by trapping the dielectric particle with the help of optical tweezers. Developed by Arthur Ashkin, a highly focused laser beam is used for micro- or nanoparticle manipulation using optical forces which can be sorted out according to their shape and size, polarizability and refractive index [76]. However, its low-throughput characteristic is the major downside of employing this approach to isolate CTCs.

Label-based methods of CTC separation includes immunoaffinity-based techniques in which specific antigens are considered that are expressed on CTC surface and separation is achieved by using specific antibodies to target those antigens. The cells can be captured by either positive or negative enrichment technique, i.e., using antigens expressed in CTCs or antigens expressed in other blood cells. Immunocapture, immunomagnetophoresis and immunofluorescence are some of the methods employed in CTC isolation using a microfluidic chip. In the immunocapturing method, CTCs are bound with antibodies for CTC isolation. EpCAM-specific antibodies or combination of antibodies (EpCAM, HER2, and EFGR) are widely used in breast, lung, prostate, ovarian, colon and renal carcinomas [77]. A negative immunocapturing approach was employed as well in which enrichment is achieved by depleting white blood cells (WBCs) with an anti-CD45 antibody [78]. Various approaches, including the construction of a micropost array [79], or employing chaotic mixing [80], geometrically enhanced mixing [81] and wavy herringbone pattern structure [82] help in improving immunocapture isolation. Similarly, in the immunomagnetophoretic approach, magnetic microparticles or nanoparticles are utilized in certain antibodies to bind with CTCs in this technique. Hoshino et al. [40] used magnetic nanoparticles linked to anti-EpCAM antibodies to perform immunomagnetophoresis separation on a blood sample containing spiking cancer cells. At a flow rate of 10 mL/h, 5 cells/mL blood were isolated. CTC enrichment can also be achieved using negative magnetophoretic isolation, which involves removing WBC from blood and using magnetic nanobeads coated with anti-CD45 antibodies. Additionally, immunofluorescence is a biomolecule isolation technique that uses antibody’s antigen specificity to target fluorescence dyes to specific biomolecules, allowing the imaging of the target molecule’s distribution. The component tagged by fluorescence-conjugated antibodies for CTC detection and the component for detected CTC isolation make up microfluidic fluorescent-activated cell sorters (FACS).

#### 2.2.2. Microfluidic Devices for Studying Cancer Cell Phenotype

The physical and biomechanical aspects of cells have long been studied, and microfluidics technology allows for a faster throughput (>100 cells s^−1^) than traditional approaches such as atomic force microscopy.

Tumor cells have mechanical properties such as stiffness and constraint, which are associated with the metastatic process, in which cells migrate through blood capillaries or lymphatic arteries to establish a colony at a secondary site. Cancer cells, according to studies, are more deformable than healthy cells [83] due to their disordered and less filamentous cytoskeletal network. Because tumor cells may easily squeeze through ECM and intravasate into the blood stream, their mechanical qualities influence not just migration but also the degree of metastasis. As a result, research into the cancer phenotype is required to understand the progression of metastatic dissemination.

Because it comprises small microchannels that imitate the movement of cancer cells in the circulatory system to distant places, the microfluidic device is the greatest tool for studying the metastatic process and analyzing the biomechanical aspects of cancer cells. Just like in the metastatic process, the cells must pass via the small pathways.

Hou et al. [41] were the first to use a microfluidic device with a straight channel and two reservoirs to assess the deformability of benign (MCF-10A) and nonmetastatic (MCF-7) breast tumor cells, evaluating quantitative characteristics such as cell velocity, cell transit time, and cell deformation. The transit velocities of two different cell lines were found to be similar, showing that cell type has no effect on transit velocity and that friction faced by cells of various stiffness may be equivalent due to smooth BSA-coated microfluidic walls. However, the entry time of MCF-10A is longer than MCF-7 cells despite being of a similar size which suggested that the reason is due to the deformability of cells and MCF-10A is stiffer than MCF-7 cells. Adamo et al. [42] presented a high-throughput system that takes advantage of the relationship between cell stiffness and travel time through small channels. Using HeLa cells and cells made compliant with Latruncilin A and cytochalasin B, they discovered that stiffer cells have a longer transit time and that cell size has a substantial impact on journey duration.

Mechanical forces generated by cells play an important role at both the cellular and tissue levels. The inability to do so causes a variety of complications, and it also provides a phenotypic basis for evaluating the diseased status and a therapeutic target in diseases such as cancer. Pushkarsky et al. [43] developed an integrated biosensor material with fluorescently labeled elastomeric contractible surfaces (FLECS) that can be used to quantify single-cell force. The device created a wide range of measurable force-generating behaviors by simulating various tissue conditions. For the cell phenotyping study, Yang et al. [44] presented harmonic acoustics for noncontact, dynamic, selective (HANDS) particle manipulation by applying time-effective Fourier synthesized harmonics for the control of particles. They used the platform to quantify cell–cell adhesion forces among various cancer cell lines and found that the adhesion strength is decreased as the metastatic potential is increased. Augustsson et al. [45] performed mechanical phenotyping of single cells using iso-acoustic focusing in the microfluidic platform. Cells flowing in a microchannel flow sideways due to the acoustic field until the point in which acoustic contrast between the cells and the medium is zero. They provided the iso-acoustic focusing of cell lines and leukocytes, showing that acoustic properties provide phenotypic information independent of size.

#### 2.2.3. Microfluidic Devices for Studying Shear Stress

The fate of tumor cells is controlled by numerous aspects once they enter the circulatory system. One of these is shear stress. Shear stress occurs when neighboring layers of fluid (blood) and viscosity move at different speeds, influencing the translational and rotational motion of CTCs and perhaps causing the deformation of CTCs, making them vulnerable to physical damages of fluidic force and immune cell attack. In arterial circulation, the fluid shear stress encountered by cancer cells is 0.4–3 Pa and 0.05–0.4 Pa in venous circulation [84]. Extravasation is aided by the adhesion of cancer cells to endothelial cells caused by fluid shear stress [85]. Mathematically, fluid shear stress = shear rate × fluid velocity. Fluid shear stress has a role in several cancer-related events, including tumor cell death, tumor development, and metastasis, as well as interactions with other blood components [86,87,88,89].

The emerging in vitro microfluidic systems can reproduce the fluid dynamical features of cancer cell in the circulatory system. For isolating and characterizing the biophysical response of single breast cancer cells to circumstances experienced in the circulatory system during metastasis, Landwehr et al. [46] used a microfluidic technique. Due to the increased length and degree of FSS, the cells were more deformable. Regmi et al. [47] created a microfluidic circulatory system to evaluate the effect of high shear stress to simulate workout conditions, and found that high fluid shear stress (60 dyne cm^−2^) eliminates more CTCs than low shear stress (15 dyne cm^−2^). High shear stress killed more than 90% of tumor cells in about 4 h and lasted for another 16–24 h. In an another study, Regmi et al. [49] discovered that ROS-generating drugs such as doxorubicin (DOX) and cisplatin can destroy the CTCs than non-ROS-generating drugs such as Taxol and etoposide under pulsatile fluidic conditions present in the bloodstream. Li et al. [48] exposed human glioma cells (U87 cells) in a straight microfluidic channel to fluid shear stress (0.12, 1.2, and 1.8 dyne cm^−2^) and found changes in cellular morphology and locomotive behavior from crawling to rolling. Fluid shear stress-stimulated cells had higher adhesion strength than nonstressed cells, while nonstressed cells had higher nuclear stiffness than cortical stiffness. The mechanical reactions of the CTCs to fluid shear stress were revealed by these findings. Marrella et al. [50] recently developed a new microfluidic device capable of simultaneously reproducing different hemodynamic wall shear stress in order to investigate the correlation with CTC cluster behaviors. Three physiological shear stress levels of 2, 5, and 20 dyne cm^−1^ were generated, simulating values of capillaries, veins, and arteries. After six hours of circulation, CTC clusters injected at these three shear stress levels caused cluster disaggregation.

#### 2.2.4. Microfluidic Device for Studying Metastasis

Cancer metastasis is responsible for 90% of cancer patient deaths [90]. Simply put, metastasis is the spread of cancer cells from the primary site to different organs. Angiogenesis, proliferation, dissociation, intravasation, circulation, extravasation and secondary tumor growth are involved in the metastatic process. The invasion sequence of cancer cells undergoes a series of steps, i.e., cancer cell development, extensive growth and invasion to basement membrane into the surrounding tissue, migration through ECM, interaction with vascular cells, invasion into blood vessels, arrest and extravasation at the secondary site and invasion into secondary tissue. Even though about 0.001–0.02% of cancer cells infiltrate the circulatory system, once metastasized, there is a substantial rise in patient mortality [91].

The epithelial–mesenchymal transition (EMT), which is promoted by tumor-associated stroma and the hypoxic environment, allows tumor cells to shed from epithelial cell–cell junction [92]. Other cells in the tumor microenvironment (TME), proteases, signaling molecules, and environmental variables around the tumor and accompanying vasculature are all elements that lead to tumor cell intravasation [93]. These cells and chemicals are critical in allowing tumor cells to infect the circulatory system through the basement membrane, attach, and pass-through endothelial cell junctions. Tumor cells must overcome blood flow shear stress and the immune system to implant at a secondary location and produce distant metastasis. Survivor cancer cells cling or are physically trapped in small capillaries at the secondary site, extravasate out of vessels, damage the ECM, and proliferate and produce a secondary tumor.

Conventional experimental models to study the metastatic process such as 2D and 3D in vitro models lack realistic complexity and so cannot accurately model the TME, metastatic process and therapeutic response. Therefore, microfluidics has emerged as a powerful platform for research and applications on oncology due to their microscaled structures which offers a high-throughput screening and ability to mimic the physiological environment.

Microfluidic platforms have been developed by many researchers to study cell invasion, intravasation, extravasation and angiogenesis which are able to reproduce biomechanical, biophysical, and biochemical microenvironments. These cues are provided to the tumor cells by ECM, mechanical stimuli, signaling molecules, noncancerous cells and soluble factors [94].

Difficulty in establishing a precise TME has rendered the study of invasion and intravasation in in vitro models challenging and crucial steps are involved during these metastatic processes of extravasation of adhesion of tumor cells to endothelium migration. Nonetheless, many novel microfluidic platforms have been developed by researchers to study metastatic cascade. Different from 2D models, these devices take cell–cell, structural and mechanical interactions into account.

Microfluidic technology could be extremely useful in cancer research. Because metastatic spread happens in a succession of phases that are typically difficult to resolve in the laboratory, they can be utilized to research the metastatic cascade. The imaging approach used in the investigation of metastasis necessitates specific knowledge and equipment, and due to their depth, a high image resolution is difficult to attain in some inner organs [95]. Each stage of the metastatic cascade, including the epithelial–mesenchymal transition, invasion, intravasation, CTC transport in the bloodstream to a secondary site, extravasation, and metastatic colonization at a distant organ, has a promising therapeutic potential for which several microfluidic assays have been developed. The interaction of cancer cells with blood or lymphatic vessels is known as the metastatic cascade. Intravasation, for example, involves cancer cells crossing the endothelium into the bloodstream, while extravasation involves cancer cells crossing the endothelium into the surrounding tissue. In microfluidic models of metastasis, the endothelium is a critical component of the microenvironment in which endothelial monolayers are used in some research.

In the process of the epithelial–mesenchymal transition (EMT), stimulants such as TGF-β1 cause epithelial cells to lose their cell polarity and cell–cell adherence, resulting in invasive and metastatic characteristics. By setting up the device on the cells that had grown in culture dishes and producing a stable concentration gradient of TGF-β1 with negligible shear stress, Kim et al. [51] were able to replicate the EMT in human lung alveolar epithelial cells (A549) in response to TGF-β1 gradients, thus providing a suitable environment for the anchorage-dependent cells. Kuo et al. [52] created a microfluidic device to study the characteristics of EMT in the microenvironment, and found that epithelial cancer cells can grow to a tumor spheroid in the device, adopt mesenchymal cell qualities, and have greater migratory capacity when compared to native cancer cells.

Additionally, many microfluidic designs are used for the study of the metastatic cascade of invasion, intravasation and extravasation. For example, Shin et al. [53] created a microfluidic chip comprising two parts: an intravasation compartment for the 3D culture of cancer cells using Matrigel matrix, and an extravasation compartment for the identification of metastasized cancer cells using adhesion molecules expressed by epithelial cells. They conducted the research to investigate a distinct component of invasion by analyzing metastatic and nonmetastatic cell lines, as well as inhibiting cancer cell invasion with inhibitors. Lee et al. [54] designed a microfluidic system that replicated cancer angiogenesis and inhibited it using anti-vascular endothelial growth factor treatment in a metastatic chip. In addition, tumor intravasation and its regulation by tumor necrosis factor–α treatment were addressed. Nagaraju et al. [55] created a three-dimensional microfluidic device with concentric three-layer cell-laden hydrogels to study breast cancer invasion and intravasation, as well as vascular development as a result of tumor vascular interaction. Kuhlbach et al. [56] developed a microfluidic platform to study extravasation that consists of two microchannels with a permeable layer sandwiched between them, with the upper channel membrane implanted with endothelial cells from the lung artery and the lower channel acting as a reservoir to accumulate extravasated tumor cells. This device can be used to study cancer cell extravasation as well as the mechanisms of rolling, adhesion, and transendothelial migration of metastatic cells.

## 3. Microfluidic Device in Anti-Cancer Drug Screening

Cancer has been a global problem and there is an urgent need to develop effective anti-cancer drugs. About 97% of oncology preclinical trials has failed to achieve the FDA approval as 2D and 3D in vitro and animal in vivo platforms has not been able to mimic the tumor microenvironment. Microfluidic platform offers numerous capabilities that mimic the TME conditions such as dynamic fluid motion, tissue–tissue interaction, spatio-temporal nutrient diffusion, inter- and intracell interactions and others which can assist in understanding the cancer cell biology and therapeutic testing.

Many factors should be considered when designing a microfluidic platform for the screening of anti-cancer drugs. Firstly, the biocompatible materials should be used for the fabrication of the microfluidic chip. Silicon or glass materials used for the fabrication have their drawbacks on high cost, opacity (of silicon) and poor permeability which are overcome PDMS-based chip due to its transparency, low cost, gas permeability and tunable mechanical properties. Still, PDMS has its issue on hydrophobicity which can bias the results in drug screening. Hydrogel has also been used to recreate the TME due to its hydrophilic nature and permeability and has a huge potential for an anti-cancer drug screening platform. Next, considerations should be made to mimic the TME on the chip. The most recent technology of tumor-on-a-chip has emerged as a new tool which provides a unique approach for understanding cancer biology and allowed cost-effective drug discovery platforms. The tumor-on-a-chip platform can accurately mimic the complex TME which consists of a mixture of many cell types such as tumor cells, immune cells, stromal cells and ECM matrix and vascularization components.

The advent of microfluidic technology has linked the pharmaceutical research by providing a novel culturing platform that can be continuously perfused and mimic the physiological functions of the tissues and organs. With the use of a lower reagent volume, it can generate mechanical stimuli such as shear stress and a well-controlled concentration gradient of molecules. Additionally, its miniature size allows for parallelization, which is essential for high-throughput drug screening.

### 3.1. Drug Response Studies Using Droplet Microfluidics

**Figure 8:**
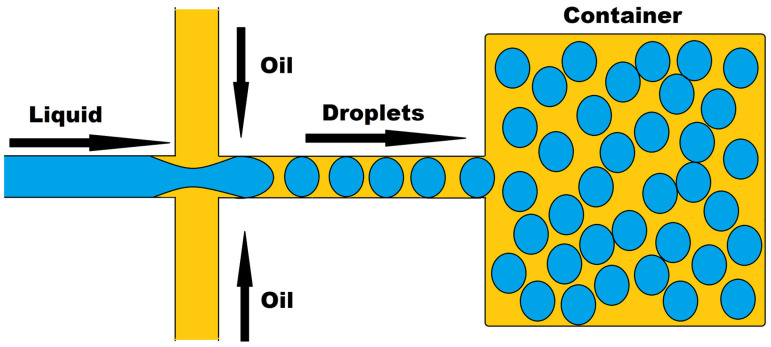
Droplet-based microfluidics.

Droplet microfluidics, a new platform that allows for the precise confinement of single cells or molecules within microdroplets for high-throughput analysis, has a lot of promise in drug development and genomics. Two immiscible liquids in a channel use confinement and capillary forces to generate these droplets, as shown in Figure 8. Thousands of cell-based assays can be performed in a single experiment with this technique, making it an excellent tool for biomedical research. Microdroplets with volumes ranging from μL to fL can be produced at very high frequencies ranging from Hz to KHz, encapsulating biomaterials such as cells, DNA, mRNA, and bacteria. Microdroplets can also be handled for mixing, combining, diluting, dividing, and sorting. It uses a smaller number of cells per device and per experiment, which may be acceptable for tumor samples with a limited number of cells. Many recent improvements in oncology have been made by combining droplet microfluidics with nucleic acid analysis [96,97], protein analysis [98], and drug discovery [99].

Droplet microfluidics, a potent technology, has been used to conduct research on single-cell-based high-throughput drug resistance investigations and drug screening for therapeutic purposes. These droplets can be utilized to encapsulate anti-cancer drugs as well as other molecules such as antibodies, proteins, growth factors, indicators, and macrophages for a variety of bio-applications, and they can be released by active or passive processes. These droplets can be created using microfluidic techniques, allowing for programmable drug absorption, confinement, and controlled release [100].

Brouzes et al. [57] demonstrated a droplet-based microfluidic system that allowed for the encapsulation of a single cell and reagent in separate aqueous microdroplets that were individually labeled with an optical label. The droplets were then combined and tested for cytotoxicity against U937 cells. Yu et al. [58] exploited the benefits of a droplet-based microfluidic system to make alginate beads with entrapped breast tumor cells for drug testing utilizing tumor spheroids rather than single cells, and found that tumor spheroids responded to doxorubicin in a dose-dependent manner. Wang et al. [59] created a microfluidic droplet-based approach for forming multicellular tumor spheroids utilizing alginate and Matrigel mixed hydrogel beads while containing HeLa cells and observing a dose-dependent response to vincristine. Sabachandani et al. [60] devised a microfluidic device to produce 1000 individual 3D spheroids for the sequential treatment of doxorubicin and paclitaxel.

The platform of droplet microfluidic technologies has been proven to be ideal for anti-cancer drug screening because to its advantages of low drug quantities, high-throughput data collection, and low cell numbers in this research. Furthermore, the elimination or reduction in cell processing stages prior to application to the droplet microfluidic device transforms it into an entirely automated high-throughput platform [101].

### 3.2. Organ-on-a-Chip Platform

Drug development has always been a field which requires novel, innovative, and insightful methods for the testing of the new drugs or molecular compounds to find the beneficial effects against many diseases. Before testing it on humans, researcher must conduct much preclinical research in vitro or in vivo to provide the detailed information about dosing and toxicity levels. The costly animal testing methods of drug testing have been there for many decades and is still existing. Animal models have also immensely contributed to our understanding of physiology and drug response; however, the efficacy and toxicity in animals and humans might not be in accordance and might not be as predictive as one might think [102,103]. Before conducting the animal testing, all the testing compounds should be studied using 2D or 3D cell culture models. In 2D cell culture systems, cells are grown on a flat dish which carries some inherent flaws of cell–cell signaling. Three-dimensional cell cultures have shown improvements in the studies of morphology, cell number monitoring, proliferation, response to stimuli, differentiation, drug metabolism, and protein synthesis and have been a more accurate representation of the in vivo scenario [104]. However, 3D cell cultures techniques fail to recreate the crucial features of the living organs such as tissue–tissue interface, concentration gradients and mechanical simulations of the microenvironment. Therefore, despite the development of cell culture techniques, currently more than 80% of drugs fails on clinical testing [105].

To address these drawbacks of the in vitro approaches, the microfluidic platform of “Organ-on-a-chip” prevailed to imitate the activities, mechanics and physiological response of the organ and provided a bridge between in vivo and in vitro practices. Organ-on-a-chip is a biomimetic, microengineered device that mimics essential physiological organ properties such as concentration gradient, shear force, cell patterning, tissue boundaries, and tissue organ interactions [106]. A single-cell type is lined in a microchannel or planted in a single chamber while being perfused with media in the simplest model of organ-on-a-chip. Donald Ingber created the first organ-on-a-chip to mimic the human breathing lung, which included alveolar epithelial cells of the lung interacting with endothelial cells within a microfluidic channel that mirrored the structure and function of tissue-vasculature structure of the alveoli of the lung [107].

The development of cytology, tissue engineering, and microengineering as an alternative to understand the physiology and drug development process has resulted in organ-on-a-chip development. The microfluidic platform offers a significant advantage in terms of precise fluid control via external valves and the creation of concentration gradients to mimic the key structure and functions of tissues and organs. This technology is also more cost-effective since it uses a less time-consuming in vitro procedure and reduces fabrication expenses. The fundamental benefit of adopting a microfluidic platform is the laminar fluid flow, which prevents nearby streams from mixing. This unique property is being used to investigate cell motility in response to chemical stimuli, as well as other sophisticated cell activities.

Three-dimensional arrangements of tissues on platforms, the incorporation of multiple types of cells to represent a more physiological balance of cells (such as stromal, vascular and immune cells), and the existence of mechanical influences (such as shear stress) appropriate to the tissue being designed are all defining characteristics of organ-on-a-chips [102]. To effectively mimic the biological milieu in the chip, one must be mindful of the flow dynamics that microfluidic technology can produce. Depending on the flow conditions, the cells in the body are subjected to continual mechanical forces or shear forces. To simulate the microsystem, the oxygen rate, nutrition rate, and blood flow rate must all be precisely controlled.

**Figure 9:**
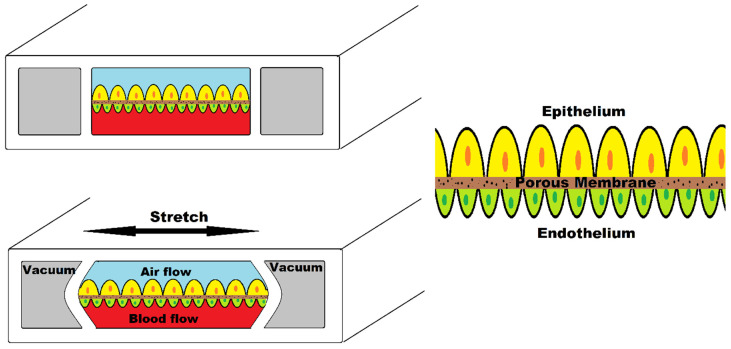
Human lung-on-a-chip device.

Figure 9 illustrates human lung-on-a-chip device simulating breathing mechanism. This device consists of two central chambers and two side chambers. The central chamber is separated by a porous membrane; the upper part is seeded with alveolar epithelial cells and the lower part contains vascular endothelial cells. Two side chambers simulate the breathing mechanism when vacuum is applied by stretching the membrane.

Hassell et al. designed such cell culture technology injecting non-small cell lung cancer (NSCLC) cell line within primary alveolus to recapitulate the tumor growth, tumor dormancy using mechanical breathing actuation [108]. They also observed the tumor response to tyrosine kinase inhibitor (TRI) therapy. Such mechanical forces to replicate the tumor microenvironment have been used by Strelez et al. who developed colorectal-cancer-on-chip device to study early invasive stage of cancer [109]. Peristalsis-like cyclic stretching mechanical forces and fluid flow are incorporated in a chip consisting of endothelial and epithelial compartments separated by porous membrane.

Many brain, lung, heart, kidney, artery, skin, bone, and cartilage organ-on-a-chip models have been presented that can imitate the activities and physiological responses of the full organ. The basic physiological requirements for the specific organ, as well as critical elements such as cell kinds, structures, and the organ’s specific physiochemical environment, should be examined, and a cell culture system should be devised based on these qualities.

The bioprinting technology relies on layer-by-layer printing, which has been used in organ-on-a-chip fabrication, wherein multiple bio functional components and cell types can be printed onto a surface of cell-compatible biomaterials to create a 3D complex structure with high spatial resolution and reproducibility [110]. Three-dimensional bioprinting on organ-on-a-chip allows for a multiscale setup of cells or biological molecules that is similar to their original microenvironment, allowing for physiological interactions under diverse conditions which better capture tissue environmental factors [111]. The bioprinting approach has a great deal of potential for organ-on-a-chip devices since it can increase tissue and function by organizing precise cell arrangements and speed up medical research [112].

### 3.3. Organ-on-a-Chip in Cancer Research

The convergence of numerous fields such as biology, physics, and engineering has resulted in new advancements in replicating physiological systems of the body, which have opened new opportunities for researching various aspects of human physiology and the drug development process [106]. Organ-on-a-chip is a cutting-edge technology for simulating organ function at the cellular level. Such platforms can be utilized to accurately imitate the microenvironment and model different disorders of the neurological system, respiratory system, digestive system, cancer, and others by incorporating biophysiochemical variables. Microfluidics and the organ-on-a-chip platform in the pharmaceutical industry can be regarded a paradigm shift in the drug development process for disease modelling and drug testing and show a great potential for revolutionizing the drug development process [113].

Microfluidics and organ-on-a-chip technology can be extremely beneficial in cancer research. The fundamental problem is a lack of understanding of the tumor microenvironment’s diversity and heterogeneity. Traditional in vitro systems have made a significant contribution to our present knowledge of the disease and treatments. However, due to a lack of structural, mechanical, and biological cues, these lack the genuine intricacy of cell-ECM and cell–cell interactions. Mechanical factors such as fluid shear stress, hydrostatic pressure, and tissue deformation were missing in complexity in the 3D tissue structure of living organs. They were also not perfused with nutrient-rich media running via an endothelium-lined vascular, resulting in a lack of understanding of tissue–tissue interactions, the involvement of circulating immune cells, and therapeutic drug physiological dosage. Organ-on-a-chip is a new platform for cancer research that is based on the microenvironment of a specific organ in which key molecular, biophysical, cellular, and tissue elements can be differed in a controlled fashion to better understand the pathophysiology of cancer in a target organ.

The organ-on-a-chip platform have a lot of potential in cancer research since they can replicate the physiological function and 3D microstructure of a human organ and simulate the organ’s complexity. They offer a cost-effective high-throughput test option with good replicability. Tumor-on-a-chip is an appealing promise of organ-on-a-chip for studying cancer biology and expedited therapy possibilities for cancer cells.

### 3.4. Replication of Tumor Microenvironment on Chip

Cancer cells, stromal cells, immune cells, and vascular cells are all lodged in the tumor tissue. Dense extracellular matrix (ECM) components are also present. An effect of cancer stromal interaction on carcinogenesis, angiogenesis, tumor invasion, metastasis, and treatment resistance has been discovered. Tumor growth is influenced by molecules released by malignant and stromal cells. Immune cells, too, have a role in tumor growth. The three-dimensional nature of solid tumors has a significant impact on tumor dynamics. As a result, cancer can be defined as a collection of complex diseases, necessitating the development of tumor models based on their three-dimensional nature and structural and dynamical complexity.

Only by considering the features of the actual tumor, including biophysical and metabolic factors, can a realistic tumor be represented. Cancer initiation, progression, and treatment effects are all influenced by the tumor microenvironment. TME’s cellular and noncellular components communicate bi-directionally to control cancer cell proliferation, function, and death. The release of soluble substances in the interstitial fluid, cell–cell or cell-ECM adhesion, and mechanical aspects all play a role in cell signaling. Shear stress, fluid forces, interstitial flow, ECM organization, composition, and stiffness are all mechanical forces.

ECM components play a key role in all cellular activities, including cell signaling and cell migration, as well as providing mechanical stability in the microenvironment. Hypoxia inducible factor (HIF-1), a transcription factor that responds to a decrease in available oxygen in cells, is one of the chemical alterations found within the tumor niche. Because the tumor lacks access to blood vessels, cells enter a condition of hypoxia, and HIF-1 accumulates quickly, driving angiogenesis by allowing vessels to develop in the TME. Alterations in DNA, such as the mutation in the p53 transcription factor, can potentially cause physiologic changes. p53 is a tumor-suppressor protein that can block cell cycle progression in G1 phase to allow damaged DNA to be repaired before DNA replication. p53 can also induce apoptosis in cells if their damaged DNA cannot be fixed. A loss of p53 gene function will allow cells with damaged DNA to be replicated, which can promote tumor formation [114].

Advanced microfabrication and tissue engineering techniques can be applied to microphysiological systems to attain physiology in the microscale, allowing clinical adaptations of preclinical findings in vitro to be facilitated. Microfluidic tissue chips are frequently made using lithographic techniques and replica molding. These chips can help imitate physiological flow, shear stress, nutrient delivery, and medication exposure by manipulating fluid. The fluid flow dynamics may be easily simulated mathematically thanks to laminar flow of fluid at the microscale, allowing theoretical predictions of complicated biological events.

Although a full regeneration of TME can be challenging, researchers have recapitulated some key features of TME over the years including vasculature, co-culture, shear stress, pressure, and chemical and oxygen gradients. A study was recently conducted by Bhattacharya et al. on the role of oxygen in tumor–immune interactions in which breast cancer cell were grown in 3D scaffolds to generate physio- and pathophysiological oxygen levels [115]. Tumor-on-a-chip platforms can very well recreate human TME and hold a great promise for cost-effective and high-throughput drug screening platforms.

Regarding tumor–stromal interactions, it plays a pivotal role in tumor growth progression along with metastasis and therapeutic intervention. These nonmalignant stromal cells consist of ECM components, fibroblasts, macrophages, and endothelial cells. These cells communicate with the cancer cells by secreting a range of molecules to influence tumor behavior via several signaling pathways. These interactions modulate tumor growth, metastasis, angiogenesis, and therapeutic resistance. Several microfluidic co-culture platforms have been designed to study and investigate these interactions. Menon et al. [61] developed a microfluidic co-culture device where two cell models, bone marrow stromal cells (HS5) and liver tumor cells (HuH7) were cultured in different hydrophilic compartments where the hydrophobic compartment placed in between acted as a barrier which was later removed after cells reached confluence by introducing liquid, giving a medium for cell migration and interaction. The findings of this paper revealed tumor-induced stromal cell death, which could be triggered by the paracrine signaling of reactive oxygen species (ROS) generated by tumor cells in the main local microenvironment homeostasis. However, when compared to monoculture, tumor cell movement appeared to be aided in co-culture. Gioella et al. [62] modelled breast cancer in a microfluidic chip with both epithelial and stromal tissues for the in vitro mimicking of stromal activation in tumor epithelial invasion as the activation and morphological changes in tumor stroma has its role in tumor progression.

In addition, the ECM components embedded in the in vitro microenvironment create a 3D structural framework to provide biochemical and structural support to the cells. The ECM has been found to simulate the development of cancer cells in vitro; hence, their interaction plays an important role in TME. Synthetic scaffold-based culture methods are widely used to recreate the ECM on chip as they closely resemble natural ECM [116]. Rijal et al. [63] developed a reconstitute tissue matrix scaffold system which was fabricated using native tissue ECM whose structural and compositional feature promote cell survival, proliferation, migration, and invasion in culture along with vascularized tumors.

### 3.5. Modelling Angiogenesis

The formation of new blood vessels from existing vasculature for the supply of oxygen and nutrients to the tumor cells is known as angiogenesis. Being one of the prominent landmarks of TME, it indeed needs to be engineered within the tumor for recapitulation of tumor physiology especially when tumor exceeds the diameter greater than 200 μm as when the critical diameter of tumor exceeds this value, hypoxia develops and complex metastatic cascade signals the start of vascularization [117].

The co-culture of cancer and endothelial cells has been a way of enabling vascularization. Nashimoto et al. [64] constructed such a vascular network on tumor-on-a-chip platform by culturing tumor spheroid where human umbilical vein endothelial cells (HUVEC), human lung fibroblast (hLF) and human breast cancer cell (MCF-7) were grown. Here, the tumor spheroid’s fibroblasts triggered angiogenesis and formed a perfusable vascular network in the spheroid. Furthermore, the findings of the literature highlighted the significance of flow in the vascular network for assessing tumor activity in drug screening platform.

### 3.6. Modelling Vascularized Tumor Models

One of the diverse factors of the TME is the vascular constructs within it. The microvascular engineering method has made it easier to rebuild tumor vasculature for network analysis and anti-cancer medication testing using patient-derived tumor cellular activities. This self-organization mechanism has also been used to mimic metastatic cascades, such as tumor cell extravasation [118].

Recent development provides the progress towards bioprinted vascular networks as well. Cao et al. [65] developed such a device in microfluidic chip pairing blood and lymph vessel which were separately bioprinted with individual permeability equivalent to their native profiles. They investigated anti-cancer drugs in different combinations of blood and lymphatic vessels to analyze their dynamics and interactions in anti-cancer medication administration.

The example of engineered vascular network includes the work done by Mannino et al. [66] who created a lymphoma-on-chip model to recapitulate the interaction between immune cell, cancer cell and endothelial cell in TME. They used stainless steel wire for creating a vascular network having an average channel diameter of 32 ± 51 μm and the platform building was done using common laboratory materials and easy techniques for fabrication.

For various reasons, the integration of vascularization in tumor chip is a game changer including recreating the structure and function of vascular tumor mass, simulating crucial phages of metastasis of interaction between tumor, endothelial cells and stromal cells, building physiologically selective barriers to nutrition and medication delivery to tumor and such setup can be used to directly evaluate medicines with anti-angiogenic and anti-metastatic properties [119]. However, the development of biomaterials to replicate the human vasculature more closely is still a problem.

### 3.7. Metastatic Cascade in Organ-on-a-Chip

Cancer cells or CTCs spreading through blood or the lymphatic system to develop new tumors in distant parts of the body define the phenomenon of metastasis. This is characterized by a wide range of sequential mechanisms including invasion, intravasation, circulation and extravasation at appropriate metastatic locations. Being a leading cause of cancer-related death, the phenomenon of metastasis is subjected to a lot of research but still our understanding of disease and its path to metastasis remains limited and its linkage to the drug resistance mechanism is unclear.

Several initiatives are underway to construct a metastatic model to aid in oncology studies and the development of novel therapeutics. Years of development of microfluidic devices provided a novel organ-on-a-chip platform, a biomimetic device to recreate the essence of human tissues and organ in vitro. These devices provide realistic models for better understanding of the process of cellular dissemination and mimic TME. As a result, these are the ideal models for the pathological study of cancer as it enables researchers to investigate the tumor development, proliferation, vascularization, and sequential events of metastasis by recreating essential aspects of cell–cell and cell–ECM interactions, physical and chemical gradients and spatio-temporal hydrodynamic properties.

Skardal et al. [67] presented a device for the real-time monitoring of colon cancer cells moving from hydrogel-fabricated gut to liver constructs in a circulatory fluidic channel to study the attachment and invasion of liver, marking the first microfluidic model of metastasis to recreate the movement from 3D originating tissue to 3D target tissue. Zervantokis et al. [68] developed a 3D microfluidic platform modelling the tumor–vascular interface to link invasion with endothelial permeability relating to cytokine-induced endothelial cell activation and paracrine signaling loops involving macrophages and tumor cells. The findings reported that endothelial permeability increased by macrophage signaling or TNF-α stimulation was linked to an increased intravasation rate and blockage of TNF-α produced by macrophages reduced intravasation and restore endothelial barrier integrity. Xu et al. [69] constructed a multi-organ-on-a-chip model to study lung cancer metastasis. In this device, to simulate the lung cancer cell metastasized to the brain, bone, and liver, the bronchial epithelial, lung cancer, microvascular endothelial, mononuclear and fibroblast cells were grown on the lung chamber and astrocytes, osteocytes and hepatocytes were grown in faraway chambers. After the formation of lung tumor mass upon culture, it demonstrated EMT and invasive potential, thus producing a tool to study cell–cell interactions during cancer metastasis and replicate the in vivo milieu of cancer metastasis. Wang et al. [70] developed a model of metastasis-on-a-chip to predict therapeutic effect and dose responses of anti-cancer medications in physiologically appropriate hepatic microenvironment by simulating the growth of kidney cancer cells in the liver. This cost-effective model has proved to be feasible for screening therapeutic compounds and assessing treatment potency.

So, organ-on-a-chip devices provides a revolutionary in vitro platform to better elucidate the complex cascade of metastasis by recreating the physiochemical complexity of the disease to understand the progression of the tumor cells and the formation of the secondary colony. Furthermore, the development of personalized models of metastasis-on-a-chip will create an exact pharmaceutical screening for personalized medicine tailored to each patient.

## 4. Concluding Remarks

### 4.1. Significance of Microfluidic and Organ-on-a-Chip Device in Cancer Research

The microfluidics platform offers numerous advantages over conventional techniques including the requirement of a low amount of sample and reagents, high sensitivity, tunable flow, low cost, high-throughput screening and short processing time. The influence of microfluidic technology is seen in approaches of CTC isolation, the characterization of tumor cells through molecular diagnosis, metastatic study, and high-throughput drug screening.

Microfluidic separation techniques have the advantage of being able to handle large volumes of fluid with a high sample throughput. The use of larger sample sizes and a higher flow rate allows for new tumor diagnostic approaches and therapeutic applications.

Isolating CTCs in microfluidics by using label-based methods or label-free methods has attracted great interest and can offer a valuable insight into cancer evolution and personalized treatment. The advanced and complex metastatic mechanism can be modelled as a microfluidic platform to imitate metastatic progress in vitro, allowing the qualitative observation and quantitative measurement of the potential outcome of cancer metastasis. It helps to understand the metastatic process and physical, biological, and mechanical characteristics of CTCs. The reagents, chemical gradients and other stimuli can be precisely delivered to the cells. Additionally, the high-throughput platforms of microfluidic technology are very promising and highly attractive for rapid testing for drug and biomarker discovery.

Incredible breakthroughs in microfluidic procedures and microfluidics-based testing have been published in recent years, demonstrating the importance of microfluidics as a new approach. Microfluidic devices have been widely used in cancer research in a variety of domains. Microfluidics can build drug delivery carriers in highly regulated environments and assess the effects of encapsulated pharmaceuticals for preliminary screening to ensure their efficacy on cancer cells. Furthermore, microfluidics can mimic the characteristics of human organ-on-a-chips, allowing researchers to test the safety of new medicinal medications before clinical use. Microfluidic instruments are presently being developed and used extensively in cancer research.

Organ-on-a-chip, a microfluidic cell culture device, faithfully recapitulates the tissue- organ-level physiology which gives a novel platform for cancer cell study and drug testing. The organ-on-a-chip can revolutionize the drug development for cancer by outperforming current 2D and 3D in vitro and in vivo animal models and anti-cancer medications could be tested on this platform to gain a better understanding of the molecular mechanisms of action and toxicities before clinical trials. By realistically recapitulating patient-specific organ physiology, it has the potential to advance the field of personalized medicine as well.

Moreover, these models accurately and authentically recapitulate the TME and its essential elements such as physical and chemical gradients, cell–cell and cell–matrix interactions, making them a potent tool in battle against cancer. Tumor-associated vasculature being a critical component of TME and a promising therapeutic target, integrating microvascular network into the chip provides the close resemblance to the structure of vascularized tumor and simulates the tumor-endothelial-stromal interaction. These models offer a biomimetic environment to replicate the events of metastasis and is a potential tool for gaining insight of tumor-in-organ levels. Nonetheless, despite all the progress made, there are still several obstacles to overcome.

### 4.2. Limitations of Microfluidic and Organ-on-a-Chip Device in Cancer Research

Although the field of microfluidic looks promising in the field of oncology, we cannot ignore the problems faced in the handling of the device. The performance of microfluidic devices is dramatically influenced by air bubbles and possible clogging.

In CTCs isolation technique, analyzing large volumes of blood can significantly increase the processing time in microchannels which results in low throughput. Parallelization and automation of the process can help with this issue. Moreover, the elderly population is more prone to cancer and blood samples obtained from them are usually sticky and contain a lot of debris which results in the clogging of microchannels [120]. The label-free methods of CTCs isolation have drawbacks of low specificity resulting in the low recovery rate and purity due to the size overlap between CTCs and blood cells.

In replicating the in vivo conditions of biomimicking, stiffness and biocompatibility of material would be an issue which would affect cell culture. Thus, it is important to use materials with suitable stiffness and biocompatibility. For simulating TME, various biophysical cues should be integrated such as matrix rigidity, elasticity, and various biochemical factors in a physiologically relevant manner. In the organ-on-a-chip field, tumor-on-a-chip is still a fledgling. Future researchers should further consider the specific pathophysiological process in TME before the manufacture of the device. For long-term studies, maintaining cell viability, functionality and structural integrity of multiple tissues could be challenging.

The advantage of a microfluidic chip is that imaging on microfluidic chips makes it simple to evaluate cell response and tumor formation. Collecting cell samples from chips, on the other hand, is difficult. Several chips must be disassembled during the sample-collecting process, which might easily contaminate the cell culture environment. Simultaneously, samples may be damaged during the collection procedure. Some experimental procedures, such as immunohistochemistry, are hampered by this drawback. As a result, a more dependable sample collecting system for microfluidic chips is urgently needed [72].

The intricate production process, as well as the utilization of valves and pumps, may be unavailable in the lab. The most often used polymer, PDMS, has a problem with hydrophobicity, which can affect bioassay results. In addition, the users of a microfluidic system should possess knowledge that is not common among life science specialists.

Organ-on-a-chips, particularly tumor-on-a-chip, can replicate the key aspects of the tumor microenvironment, making them a revolutionary, reliable, and precise platform for cancer cell research covering tumor growth, metastasis, and therapeutic response. Yet, there are many aspects to be improved for more accurate and reliable results. Firstly, skilled and experienced researchers are required for the fabrication of microfluidic devices and the incorporation of 3D bioprinting and sophisticated tissue engineering techniques. The pathophysiological mechanism to be simulated must be investigated before starting the project. Computational fluid dynamics simulations will be of great help for the study of transport of materials through microchannels to improve the hydraulic design. Researchers should continuously explore the new materials which are biocompatible to avoid experimental errors that limit their application.

Furthermore, obtaining patient-specific cells has always been a significant challenge for the research. In order to combat the low translational rate of fundamental research findings, induced pluripotent stem cells (iPSCs) offer a variety of new possibilities. The capacity to maintain an undifferentiated pluripotent state in culture indefinitely and develop into any cell types in the human body has made the iPSC model popular in recent years [121]. In oncology research, its major application lies on modelling cancer progression, study of complex cancer genetics and drug development.

The metastatic nature of cancer requires innovation to recapitulate not only TME, but also the possible metastatic target which are connected by fluidic network for metastatic modelling. This novel insight is fulfilled by the human-on-a-chip model, which is a 3D cell culture system containing cellular aggregates to imitate multiple organs-on-a-body. This kind of sophisticated platform helps in measuring the direct impacts of the reaction of one organ on another. These chips have great prospects in the field of oncology as they can be used for studying anti-cancer drugs’ potential to fight human cancer cells and the effect on multiple organ tissues placed in a distant chamber.

## Data Availability

Not applicable.
